# The pregnancy hormones human chorionic gonadotropin and progesterone induce human embryonic stem cell proliferation and differentiation into neuroectodermal rosettes

**DOI:** 10.1186/scrt28

**Published:** 2010-09-13

**Authors:** Miguel J Gallego, Prashob Porayette, Maria M Kaltcheva, Richard L Bowen, Sivan Vadakkadath Meethal, Craig S Atwood

**Affiliations:** 1 Geriatric Research, Education and Clinical Center, Veterans Administration Hospital and Department of Medicine, University of Wisconsin-Madison School of Medicine and Public Health, 2500 Highland Avenue, Madison, WI 53705, USA; 2 Duke University, Department of Medicine, Chapel Drive, Durham, NC 27708, USA; 3 Institute of Pathology, Case Western Reserve University, 2103 Cornell Road, Cleveland, OH 44106, USA; 4 School of Exercise, Biomedical and Health Sciences, Edith Cowan University, 270 Joondalup Drive, Joondalup, 6027 WA, Australia; 5 Department of Neurological Surgery, University of Wisconsin-Madison School of Medicine and Public Health, 600 Highland Avenue, Madison, WI 53792, USA

## Abstract

**Introduction:**

The *physiological *signals that direct the division and differentiation of the zygote to form a blastocyst, and subsequent embryonic stem cell division and differentiation during early embryogenesis, are unknown. Although a number of growth factors, including the pregnancy-associated hormone human chorionic gonadotropin (hCG) are secreted by trophoblasts that lie adjacent to the embryoblast in the blastocyst, it is not known whether these growth factors directly signal human embryonic stem cells (hESCs).

**Methods:**

Here we used hESCs as a model of inner cell mass differentiation to examine the hormonal requirements for the formation of embryoid bodies (EB's; akin to blastulation) and neuroectodermal rosettes (akin to neurulation).

**Results:**

We found that hCG promotes the division of hESCs and their differentiation into EB's and neuroectodermal rosettes. Inhibition of luteinizing hormone/chorionic gonadotropin receptor (LHCGR) signaling suppresses hESC proliferation, an effect that is reversed by treatment with hCG. hCG treatment rapidly upregulates steroidogenic acute regulatory protein (StAR)-mediated cholesterol transport and the synthesis of progesterone (P_4_). hESCs express P_4 _receptor A, and treatment of hESC colonies with P_4 _induces neurulation, as demonstrated by the expression of nestin and the formation of columnar neuroectodermal cells that organize into neural tubelike rosettes. Suppression of P_4 _signaling by withdrawing P_4 _or treating with the P_4_-receptor antagonist RU-486 inhibits the differentiation of hESC colonies into EB's and rosettes.

**Conclusions:**

Our findings indicate that hCG signaling via LHCGR on hESC promotes proliferation and differentiation during blastulation and neurulation. These findings suggest that trophoblastic hCG secretion and signaling to the adjacent embryoblast could be the commencement of trophic support by placental tissues in the growth and development of the human embryo.

## Introduction

Zygotic division into a blastocyst establishes the extraembryonic tissues (trophoblast layer or outer cell mass) that support the embryonic embryoblast (inner cell mass) early in embryogenesis. Trophoblasts secrete an array of hormones [1-4], including hCG, during the migration of the blastocyst through the fallopian tube and its implantation into the endometrium. The dramatic elevation in the production of hCG by trophoblasts at this early embryonic stage (from 5 to ≥1,000 mIU/ml in the maternal serum) [2,5] signals both the corpus luteum and trophoblast [3] to synthesize and secrete P_4 _[6]. This is required for the maintenance of the endometrium, blastocyst attachment, and syncytiotrophoblast invasion into the endometrium [7]. However, the role of hCG in early human embryogenesis is unknown.

Given the close spatial localization of the developing trophoblast layer to the embryoblast, it is conceivable that trophoblast-associated hormones *directly *signal the growth and development of the embryoblast. hCG, and luteinizing hormone (LH), which shares 83% amino acid sequence homology, bind a common receptor, the LH/human chorionic gonadotropin receptor (LHCGR) with similar affinity [8]. LHCGR has been identified on all tissues studied to date (reviewed in [9,10]), although it has not been reported on hESCs.

Recent evidence from our laboratory supports trophoblastic hCG and P_4 _signaling to embryoblast-derived hESCs and includes (a) hCG markedly increases hESC expression of the adhesion and neuritogenic protein amyloid-β precursor protein (AβPP) [11], (b) P_4 _is required for nonamyloidogenic processing of AβPP during hESC differentiation [11], and (c) P_4 _signaling is necessary for human embryoid body (EB) differentiation [4]. Moreover, it is known that (hyperglycosylated) hCGβ has potent cell growth and invasion properties and acts as an autocrine factor on extravillous invasive cytotrophoblast cells to initiate and control invasion during implantation and the establishment of hemochorial placentation [12]. hCG also promotes these processes during malignancy in invasive hydatidiform mole, choriocarcinoma, and testicular cancers [12].

In this study we tested whether the trophoblastic hormones hCG and P_4 _signal hESC proliferation and differentiation. By using this *in vitro *model of early human embryogenesis, we found that hCG/LH signal via LHCGR to promote hESC proliferation and steroidogenesis (P_4 _synthesis), and that P_4 _signaling is obligatory for both EB and neuroectodermal rosette differentiation.

## Materials and methods

### Human embryonic stem cell culture

#### Propagation of human embryonic stem cells

Pluripotent H9 hESCs (passage 22 to 32; XX karyotype; also known as WA09, a National Institutes of Health registered line) were obtained from the WiCell Research Institute (Madison, WI). The research was approved by the UW-Madison Department of Medicine. Cells were plated onto irradiated mouse embryonic fibroblast (MEF) cells (1.875 × 10^5 ^cells per well; Biovintage, San Diego, CA) in six-well plates (Fisher Scientific, Pittsburgh, PA) coated with 1 ml of sterile 0.1% gelatin (Sigma-Aldrich Co., St. Louis, MO) solution. Before the addition of hESCs, MEF cells were grown in Dulbecco's Modified Eagle Media (DMEM) (Invitrogen, Carlsbad, CA) supplemented with 10% heat-inactivated fetal bovine serum (FBS; Invitrogen) and 1% nonessential amino acids (NEAAs; Invitrogen). After 24 hours of MEF plating, hESCs were plated on this MEF feeder layer and grown in the presence of DMEM-F12 media (Invitrogen) supplemented with 1% NEAA, 1 m*M *L-glutamine (Invitrogen), 0.1 m*M *2-mercaptoethanol (Sigma-Aldrich Co.), 4 ng/ml bFGF (Invitrogen), and 20% Knockout Serum Replacement (KOSR; Invitrogen). Continual propagation of cells required colonies to be enzymatically lifted with 1 ml of a sterile solution of collagenase type IV (Invitrogen) (1 mg/ml of DMEM-F12), dissected into multiple small pieces, and transferred onto a fresh MEF feeder layer every 4 to 5 days. hESCs also were grown on Matrigel (BD Biosciences, San Jose, CA), a basement-membrane preparation extracted from a murine Englebreth-Holm-Swarm sarcoma, in the presence of mTeSR1 medium (StemCell Technologies, Inc., Vancouver, British Columbia, Canada), a defined culture medium developed by WiCell Research Institute [13]. Matrigel (100 μg/ml in DMEM-F12; 1 ml) was added to each well of a six-well plate and left for 1 hour at room temperature or at 4°C overnight. hESCs were transferred onto these plates, and cells were passaged by enzymatic lifting with a sterile solution of neutral protease (Dispase; 1 mg/ml in DMEM-F12; Invitrogen), the colonies dissected into multiple small pieces and transferred onto new plates coated with Matrigel, and cultured in mTeSR1 medium. The culture medium (2.5 ml per well) was replaced every day in all these culture conditions.

#### Differentiation of hESCs into embryoid bodies

This protocol mimics the formation of the blastocyst during human embryogenesis [14]. Pluripotent hESCs (H9) grown on MEF in a six-well plate were rinsed twice with 1 ml of Dulbecco's Phosphate-Buffered Saline (DPBS, without calcium or magnesium; Invitrogen) per well. Colonies were then incubated with 1 ml of Dispase (0.5 mg/ml in DMEM-F12) at 37°C, 5% CO_2_, until the colonies detached intact while avoiding dispersing the colonies into single cells. T25 flasks (Fisher Scientific) were incubated with 5 ml of a 2% poly (2-hydroxyethyl methacrylate) (Poly-HEMA; Sigma-Aldrich Co.) solution for 5 minutes. The flask was placed in a horizontal position for 5 minutes with the cap on. This allowed optimal coating of the working-surface area of the flask. This process was repeated for each side of the flask. The caps of coated flasks were opened, and the Poly-HEMA solution was sucked off. The open flasks were allowed to remain in the hood for 1 hour to dry. After 1 hour, the surface was washed twice with DPBS, and the detached hESC colonies were cultured in 15% Characterized FBS (Invitrogen) and 85% Iscove's Modified Dulbecco's Medium (IMDM; Invitrogen) in these Poly-HEMA-coated T25 flasks and incubated under standard conditions (37°C, 5% CO_2_) on an orbital shaker (Boekel Orbitron, Feasterville, PA) with constant gentle rocking for 10 to 14 days to allow the hESCs to aggregate into cystic spheroidal structures. The Poly-HEMA coat and the constant gentle rocking prevented the adherence of these spheroidal structures to the flask.

#### Differentiation of hESCs into neural precursor cells

The protocol described later for the differentiation of hESCs into columnar neural precursor cells (NPCs) mimics *in vivo *NPC development in terms of timing and morphology [15]. *In vitro*, hESCs differentiate into columnar NPCs that organize into neural tube-like rosettes within 12 to 14 days. Considering that hESCs are equivalent to a 5 to 6 day embryo, development of the NPCs *in vitro *takes about 18 to 20 days, the time window when the neural tube forms in a human embryo [16,17].

Pluripotent hESC (H9) colonies grown on MEF in six-well plates were rinsed twice with DPBS (1 ml per well) and then treated with Dispase (1 ml of 1 mg/ml in DMEM-F12) and incubated at 37°C, 5% CO_2 _until the edges of the colonies began to curl up. The plate was then swirled to detach the colonies intact but without dispersing the colonies into individual cells. The hESC colonies were grown in T25 flasks in a special ES cell-growth medium (78.5% DMEM-F12, 20% KOSR, 1% NEAA, 1 m*M *L-glutamine, 0.1 m*M *2-mercaptoethanol) for 4 days with daily replacement of media to form ESC aggregates. ESC aggregates were then adhered to the culture surface, where they formed monolayer colonies in a chemically defined neural induction medium (32.6% F-12 (Invitrogen), 65.2% DMEM, 1% N2 supplement (Invitrogen), 1% NEAA, 0.2% of 1 mg/ml Heparin (Sigma-Aldrich Co.), and 10 ng/ml bFGF). Under this culture condition, columnar NPCs appear in the center of each colony and organize into neural tube-like rosettes after a total of 9 to 10 days of differentiation culture. The neural induction medium was replaced every other day. The NPCs in the rosettes were selectively isolated through differential enzymatic treatment by using Dispase (0.5 mg/ml in DMEM-F12) and incubated for 2 hours in neural induction medium to allow the nonneural cells to attach differentially attach to the T25 flasks. After this, the floating cells (mostly aggregates of NPCs) were transferred to new T25 flasks where they rolled up to form round clusters. Some of these clusters were collected and probed for nestin to confirm NPC differentiation. The remaining formed clusters were continuously passaged by manually splitting the clusters by using a sterile scalpel.

### Treatment of hESCs

Pluripotent H9 hESCs were plated in six-well plates coated with Matrigel in 2.5 ml of mTeSR1 medium per well. After overnight culture, cells were treated every day in different experiments with (a) hCG (0 to 50,000 mIU/ml; Ray Biotech Inc., Norcross, GA) in mTeSR1 medium for 6 days; (b) LH (0 to 100 mIU/ml; National Peptide Hormone Program, Harbor-UCLA, Los Angeles, CA); (c) P_4 _(2 μ*M*), E_2 _(10 n*M*), RU-486 (20 μ*M*; stock RU-486 was solubilized in EtOH and then diluted into media; Sigma Laboratories, St. Louis, MO), DMSO (equivalent volume to that added to sex steroids) and EtOH (equivalent volume to that added to RU-486) for 5 to 10 days in TESR1 medium (lacking Li); (d) affinity purified anti-LHCGR rabbit polyclonal antibody (1:1,000, 1:200, 1:100 dilution) in combination with hCG; or (e) lipofectamine (control), lipofectamine + LHCGR sense oligonucleotides or lipofectamine + LHCGR P-antisense oligonucleotides for 6 days. For experiments using oligomers with phosphorothioate bonds (antisense-P; Integrated DNA Technology, Coralville, IA), oligomers were added to media (240 μl) that had been preincubated with lipofectamine (4 ng/μl; Invitrogen) for 5 minutes at room temperature. This mixture was then incubated at room temperature for 20 minutes before the addition to cells. Antisense-P was used at a final concentration of 0.4 μ*M*.

Cells were counted by using the trypan blue staining method and collected in Dulbecco's phosphate-buffered saline (DPBS) and stored at -80°C before analysis of mRNA or protein expression or both. Media from hCG-treated cells were pooled each day for 6 days (15 ml total), lyophilized, and resuspended in 2 ml of TESR1 medium for EIA of P_4 _(Cayman Chemical Company, Ann Arbor, MI).

### Progesterone receptor antagonist treatment of hESC colonies

#### EB formation

ESCs were allowed to form colonies by culturing for 4 days on an MEF feeder layer, as described earlier, and then were enzymatically lifted and the colonies placed into EB medium (containing serum) in the absence (control) or presence of RU-486 (20 μ*M*) and rocked gently for an additional 10 days to allow EB formation. Structures were then assessed morphologically.

#### Rosette formation

hESCs were allowed to form colonies by culturing for 4 days on an MEF feeder layer. Colonies were then enzymatically lifted and placed into EB medium (containing serum) and rocked gently for an additional 4 days. Colonies were then placed in neural induction media without P_4 _(using an especially made medium containing all ingredients except P_4_), with P_4 _(2 μ*M*), and/or RU-486 (20 μ*M*) for an additional 11 days. At 19 days, the structures were assessed morphologically and then collected for immunoblot analyses.

### mRNA Expression

Total RNA was isolated from cultured hES cells by using the RNeasy Mini Kit (Qiagen, Valencia CA) according to the manufacturer's instructions. LHCGR, LHβ, hCG, and steroid acute regulatory protein (StAR) cDNA were synthesized and amplified by using the SuperScript III One-Step RT-PCR system (Invitrogen). Both cDNA synthesis and PCR amplification were carried out by using gene-specific primers (Integrated-DNA-Technologies, Coralville, IA): *LHCGR *forward 5' CCCTCACCGTCATCACTCTAG 3', reverse 5' CGATTTCACCTGCATGGC 3', *LHβ *forward 5' GCTACTGCCCCACCATGATG 3', reverse 5' ATGGACTCGAAGCGCACATC 3', Isoform V of *hCGβ *forward 5' ATCACCGTCAACACCACCATCTGT 3', reverse 5' AAGCCTTTATTGTGGGAGGATCGG, *StARD1 *forward 5' CGGCAGCGACCCCACCACT 3', reverse 5'AGCCGGAACACCTTGCCCACAT 3'. PCR amplification of *LHCGR *was performed for 35 cycles of 95°C for 30 seconds, 62°C for 45 seconds, 72°C for 60 seconds, and final extension time of 5 minutes. PCR amplification of StAR was performed for 35 cycles of 95°C for 30 seconds, 64°C for 45 seconds, 72°C for 60 seconds, and a final extension time of 5 minutes. PCR amplification of *LHβ *was performed for 35 cycles of 95°C for 30 seconds, 63°C for 45 seconds, 72°C for 60 seconds, and final extension time of 5 minutes. PCR amplification of *hCG *was performed for 35 cycles of 95°C for 30 seconds, 63°C for 45 seconds, 72°C for 60 seconds, and final extension time of 5 minutes. The PCR product was stained with Gel Star Nucleic Acid Stain (Cambrex Bio Science, Rockland, ME), run on 2.5% Metaphor agarose gel (Cambrex Bio Science), and imaged by using EC^3 ^Imaging System (UVP Bioimaging System, Upland, CA).

### Immunoblotting

Cells were collected and immunoblot analyses performed as previously described [11,18]. Because of the dramatic changes that occur in protein expression during the dynamic developmental period under consideration, it was difficult to find an internal control to demonstrate equal protein loading (see [11,18]). Given this variability, we chose to load samples according to total protein, as previously described [19]. The following antibodies were used throughout this study: Anti-human nestin monoclonal antibody (Chemicon International, Temecula, CA); affinity purified anti-human LH/hCG receptor polyclonal antibody generated against the N-terminal 15-38 amino acids (New England Peptides, Atlanta, GA); anti-human Oct3/4 monoclonal antibody, anti-human PR rabbit polyclonal antibody (C-19) generated against the C-terminus, glyceraldehyde-3-phosphate dehydrogenase (GAPDH) goat polyclonal antibody (V-18), anti-human β-actin goat polyclonal antibody (C-11), horseradish peroxidase-linked goat anti-mouse, goat anti-rabbit, and donkey anti-goat IgG (Santa Cruz Biotechnology); anti-human LHβ polyclonal antibody (National Hormone and Peptide Program, Harbor-UCLA Medical Center, Torrance, CA) [20]; anti-human StAR polyclonal antibody (a kind gift of Dr. Strauss, University of Pennsylvania [21]).

### Enzyme immunoassay

hESCs were treated with hCG (500 mIU/ml) in TESR1 media each day for 6 days, the media collected and pooled each day (15 ml total), lyophilized and resuspended in 2 ml of TESR1 media for enzyme immunoassay (EIA) of P_4 _(Cayman Chemical Company) according to the manufacturer's instructions.

### Statistical analysis

Statistical analysis was performed by using the Student's *t *test and ANOVA analyses followed by pair-wise comparisons with Fisher's protected least significant difference procedure (PLSD) to determine significant changes between treatment groups (Statview 5.0 & SuperAnova 3.0 programs; SAS Institute, Inc.).

## Results

**Figure 1:**
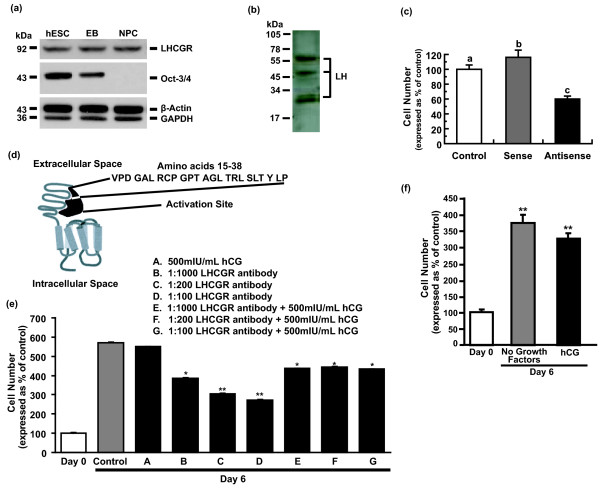
**hCG signals hESC proliferation via LHCGR**. **(a) **Pluripotent H9 hESC, EBs and NPC lysates were analyzed by immunoblot with **(i) **an affinity-purified anti-human LH/hCG receptor polyclonal antibody against the N-terminal 15-38 amino acids; **(ii) **a monoclonal antibody against Oct-3/4 (C-10; against 1-134 amino acids of human Oct-4), and **(iii) **monoclonal antibodies against human β-actin and GAPDH [11,18]. Molecular-weight markers are shown on the left of immunoblots. **(b) **Protein from cell lysates of hESCs were analyzed with immunoblot by using a polyclonal antibody against LHβ [20]. **(c) **hESCs were treated with lipofectamine (control), lipofectamine + LHCGR sense oligonucleotides, or lipofectamine + LHCGR antisense-P oligonucleotides for 6 days, and the cells were then counted by using the trypan blue method. Results are expressed as mean ± SEM, *n *= 4; significant differences between groups is indicated by different letters, *P *< 0.05. **(d) **Schematic of the LHCGR activation site and binding site of rabbit polyclonal antibody against amino acids 15 to 38 of the extracellular binding domain of LHCGR. **(e) **hESCs grown in six-well plates coated with Matrigel in mTeSR1 media were treated for 6 days with **(i) **hCG (500 mIU/ml; lane A); **(ii) **increasing concentrations of the affinity-purified rabbit polyclonal antibody against amino acids 15 to 38 of the extracellular binding domain of LH/hCG receptor (1:1,000, 1:200, 1:100; lanes B, C, and D, respectively); and **(iii) **in combination with (500 mIU/ml of hCG (lanes E, F, and G). Cell number was counted by using the trypan blue assay. Results are expressed as mean ± SEM, *n *= 3; *P < 0.05; **P < 0.005 compared with day 6 control. **(f) **hESCs were cultured in growth factor-free TESR1 medium ± hCG (500 mIU/ml) for 6 days, and cell number was measured by using the trypan blue assay. Results are expressed as mean ± SEM, *n *= 3; **P < 0.005 compared with 6 day control.

To examine the functionality of trophoblastic hCG signaling to the embryoblast, we first examined whether the LHCGR was expressed by pluripotent hESCs. Full-length mature LHCGR (92 kDa) [2] was detected in hESCs, and expression was not altered on differentiation into EB's, which recapitulate the early stages of perimplantation embryos [22], or into neuroectodermal rosettes, which consist of >90% columnar NPCs and are the *in vitro *equivalent of a rudimentary neural tube [15] (Figure 1a, upper panel). Decreased Oct-3/4 expression (Figure 1a, middle panel) together with bright-field analysis (not shown) indicated lineage commitment and loss of pluripotency during the differentiation of hESC colonies into rosettes. RT-PCR of RNA extracted from pluripotent hESCs confirmed the presence of LHCGR message (Supplemental figure S1 in Additional file 1). The comparable level of LHCGR expression between the different cell lineages is suggestive of a basal requirement for LH/hCG signaling during these early stages of embryogenesis.

Because hCG is mitogenic toward epithelial and endothelial cells of the endometrium and is a marker of carcinogenesis [23], we tested whether hCG was generated by hESCs and is mitogenic toward hESCs. RT-PCR amplification of RNA extracted from pluripotent hESCs by using sequence-specific primers confirmed the presence of both hCGβ isoform V and LHβ message (data not shown). Although full-length mature 30-kDa LH protein (α-GSU + LHβ subunits) and other variants of LH (47 kDa and 60 kDa) [20] could be detected with immunoblot analysis (Figure 1b), hCG was not detectable by immunoblot, as previously reported for hESCs [3], suggesting differential translational control of the expression of these gonadotropins early in embryogenesis. These results suggest that before hESC differentiation into trophoblasts, which are known to secrete hCG [3], LH expressed by hESCs may act in an autocrine manner on hESCs. To examine the requirement for LH/hCG signaling in the proliferation of hESCs *in vitro*, we treated hESCs with P-antisense oligonucleotides against LHCGR. P-antisense oligonucleotides significantly decreased hESC proliferation (48%) compared with sense oligonucleotide-treated hESCs (Figure 1c). To confirm that hESC production of LH or trophoblastic production of hCG promotes hESC proliferation, we treated hESCs with increasing concentrations of an antibody against amino acids 15 to 38 of the extracellular activation site of LHCGR (Figure 1d) [24]. This antibody significantly reduced hESC proliferation in a dose-dependent fashion compared with 6-day untreated controls (Figure 1e; 48% reduction with 1:100 diluted antibody). Addition of hCG to hESCs treated with this blocking antibody reversed this effect, confirming the specificity of the antibody for the receptor and of hCG signaling for hESC proliferation. Similar results were obtained with LH treatment (data not shown). Treatment of hESC with a physiologically relevant concentration of hCG (500 mIU/ml) in *growth factor-free *TESR1 culture medium resulted in a 3.3-fold increase in cell proliferation after 6 days (Figure 1f). Surprisingly, a similar increase (3.7-fold) in hESC proliferation was observed in *growth factor-free *TESR1 culture media (that is, TESR1 media containing no bFGF and TGF-β), suggesting the autocrine production of hCG/LH (or other mitogenic factors) by hESCs or hESCs that had differentiated into trophoblasts in our cultures, as previously reported by Thomson *et al. *[25]. The high binding affinities (*K*_d _≈0.4-5.5 × 10^-10 ^*M*) of hCG/LH for the human receptor [26,27] indicates that the autocrine production of even very low concentrations of these gonadotropins by hESCs (or trophoblasts, as reported by Golos *et al*., [3]) is sufficient to signal hESC proliferation. A low level of hCG/LH expression by hESC/trophoblasts also is consistent with the high binding capacity (~2.2 fmol/mg tissue) of hCG for LHCGR [26]. In this respect, knockdown of Oct4 expression in hESC induces hCG and Gcm1 expression [28] and indicates that any small differentiation of hESC in our cultures may provide sufficient hCG for hESC proliferation. Together, these results suggest the presence of an hCG/LH-dependent mechanism that signals embryonic growth.

**Figure 2:**
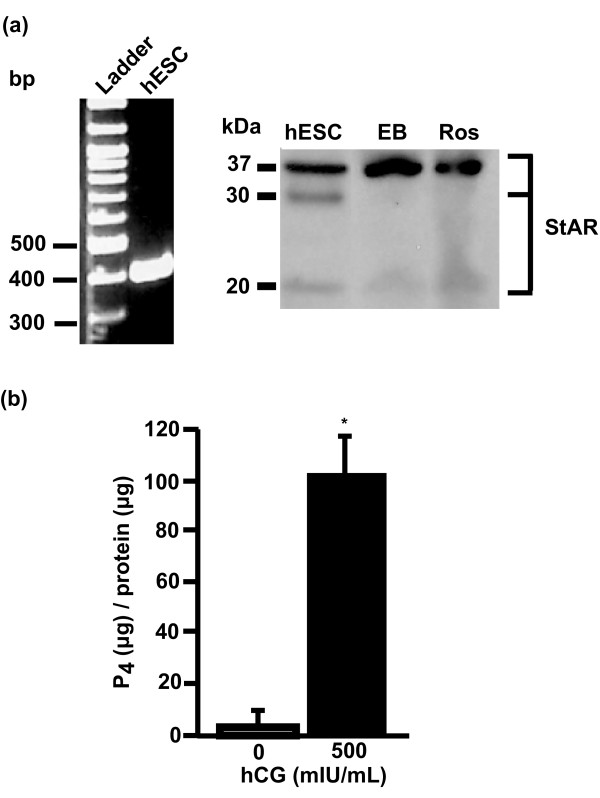
**hCG induces the synthesis and secretion of P_4 _by hESCs**. **(a) **Total RNA isolated from hESCs was amplified by RT-PCR by using two pairs of sequence-specific primers for StAR. The expected 404-bp and 408-bp cDNA fragments were detected (left panel). Equal amounts of protein from cell lysates of hESCs, EBs and rosettes were analyzed with immunoblot with an anti-human StAR polyclonal antibody [21] (right). Molecular-weight markers are shown on the left. **(b) **The concentration of P_4 _secreted into the media from hESCs treated ± hCG (500 mIU/ml) for 6 days. Results are expressed as micrograms P_4 _per microgram cellular protein, mean ± SEM, *n *= 3; *P < 0.001.

hCG functions to increase trophoblast and corpus luteal P_4 _production [6,29]. To examine whether hCG induces steroidogenesis in hESCs, as in steroidogenic tissues, we examined the expression of StAR, a key rate-limiting step in the production of steroids [30]. hESCs were found to express StAR mRNA and protein (37-kDa, 30-kDa, and 20-kDa variants; Figure 2a). Increases were found in the expression of the mature variant (37 kDa) and decreases in the expression of the truncated (30- and 20-kDa) variants of StAR with differentiation of hESCs into EBs and rosettes (Figure 2a). Because truncation of the 37-kDa to the 30/32-kDa variants of StAR is indicative of increased cholesterol transport across the mitochondrial membrane for steroidogenesis [30], these results suggest a decreased requirement for steroidogenesis in more-differentiated cell lineages.

To assess the steroidogenic potential of hESCs, we treated hESCs with hCG and measured P_4 _secretion into the media. hCG (500 mIU/ml) treatment increased P_4 _secretion into the media 15-fold (Figure 2b), suggesting that P_4 _may signal hESCs.

**Figure 3:**
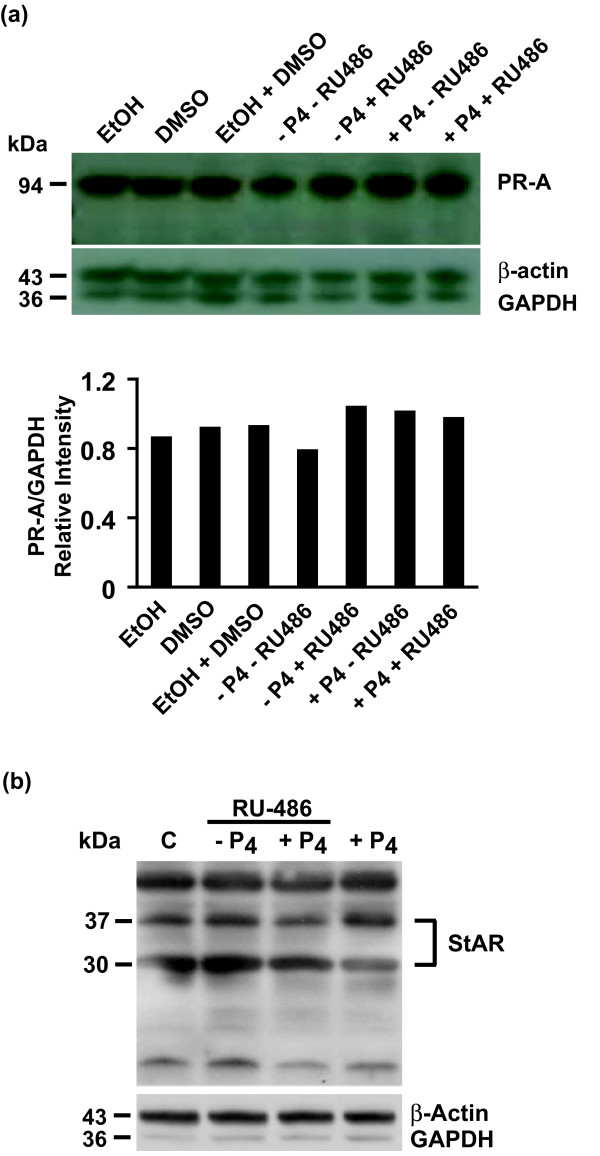
**P_4 _signals via PR on hESCs**. **(a) **hESCs treated ± P_4 _(2 μ*M*), RU-486 (20 μ*M*), and/or appropriate controls for 5 days were collected, and equal amounts of protein from cell lysates were analyzed by immunoblot for PR-A with anti-human PR rabbit polyclonal antibody (C-19) generated against the C-terminus, β-actin antibody and GAPDH antibody [11,18]. EBs were treated ± RU-486 (20 μ*M*) and analyzed for these proteins. **(b) **hESCs were treated with and without P_4 _(2 μ*M*) or RU-486 (20 μ*M*) or both for 5 days and analyzed with immunoblot for StAR, β-actin, and GAPDH. Molecular-weight markers are shown on the left of immunoblots.

Analysis of hESCs and EBs indicated that these cells express P_4 _receptor A (PR-A) (Figure 3a), as was previously reported for the mRNA [31] and murine ESCs [32], suggesting that P_4 _could signal hESC differentiation. PR-A expression did not change in hESCs after treatment with P_4 _or the PR antagonist RU-486 (Figure 3a). That PR-A is functional in hESCs is supported by the finding that P_4 _treatment decreases truncation of the 37-kDa StAR variant (increasing the 37- to 30-kDa ratio by 83%; Figure 3b). These results also indicate that mechanisms at the level of both StAR expression and processing exist to regulate hESC steroidogenesis. Together, these results indicate that negative-feedback pathways exist for the regulation of hCG/LH signaling and cholesterol uptake for the synthesis of sex steroids in hESCs and differentiating lineages.

**Figure 4:**
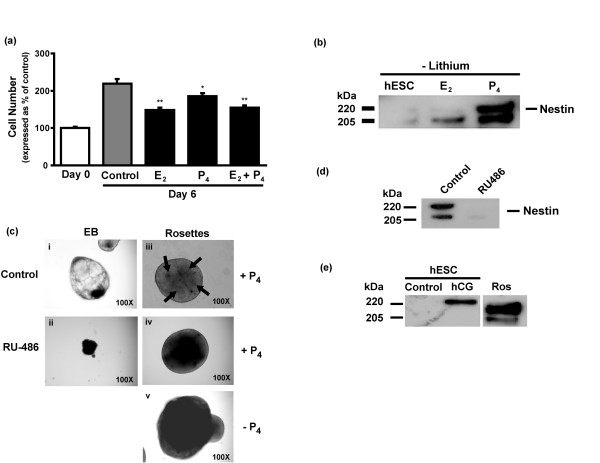
**P_4 _promotes blastulation and neurulation**. **(a) **hESCs were grown in lithium-free TESR1 medium in the presence of E_2 _(10 n*M*) or P_4 _(2 μ*M*) or E_2 _(10 n*M*) + P_4 _(2 μ*M*) for 6 days, and cell numbers were measured by using the trypan blue assay. Results are expressed as mean ± SEM, *n *= 4; *P < 0.05; **P < 0.005 compared with 6 day control. **(b) **Equal amounts of protein from cell lysates of hESCs treated for 9 days as described earlier were analyzed with immunoblot by using a monoclonal antibody against nestin (clone 10C2). **(c) **EB formation: hESCs were allowed to form colonies by culturing for 4 days on an MEF feeder layer, as described earlier, and then were enzymatically lifted, and the colonies were placed into EB medium (containing serum) in the absence (i; control) or presence (ii) of RU-486 (20 μ*M*) and rocked gently for an additional 10 days to allow EB formation. Structures were then assessed morphologically. Rosette formation: hESCs were allowed to form colonies by culturing for 4 days on an MEF feeder layer; colonies were then enzymatically lifted and placed into EB medium (containing serum) and rocked gently for an additional 4 days. Colonies were then placed in neural induction medium with P_4 _(2 μ*M*) (iii), without P_4 _(v) or with P_4 _(2 μ*M*) + RU-486 (20 μ*M*) (iv) for an additional 11 days. At 19 days, the structures were analyzed morphologically. **(d) **The structures in (c) were collected, and equal amounts of protein from cell lysates were analyzed for nestin with immunoblot analysis. **(e) **hESCs were grown in mTeSR1 medium and treated with 500 mIU/ml hCG for 8 days. Cells were collected, protein content determined by using the BCA assay, equal amounts of protein were run on SDS-PAGE, and the immunoblot was probed for human nestin. Molecular-weight markers are shown on the left of immunoblots.

Increasing concentrations of hCG suppress the pluripotent marker Oct-3/4 (Supplemental figure S2 in Additional file 1), suggesting that hCG, or steroid production initiated by hCG signaling, can direct lineage commitment. To test the effects of sex steroids on hESC proliferation and differentiation, we treated hESCs with E_2_, P_4_, and E_2 _+ P_4 _and observed a significant decrease in cell proliferation by 29%, 16%, and 23%, respectively, compared with untreated control (Figure 4a). These results were consistent with the slight decrease in cell proliferation observed after hCG treatment (Figure 1f) that induced significant P_4 _secretion (Figure 2b). A screen of germ-layer markers indicated that P_4_, and, to a lesser extent, E_2_, increase the expression of nestin, an early marker of NPC formation, in hESCs (Figure 4b). Interestingly, E_2 _'priming' has been shown to be required for induction of PR expression in other tissues [33]. Thus, the increase in nestin expression with E_2 _treatment may reflect increased PR expression together with endogenous P_4 _signaling, and explain the current requirement for serum priming of hESC colonies in the preparation of neuroectodermal rosettes. Previous studies have demonstrated the importance of P_4 _and related steroids as neurotrophic agents that promote adult neurogenesis, neuronal survival, and neuroprotection [34-36].

The induction of nestin expression by P_4 _reveals a pivotal function for this pregnancy hormone during ectoderm formation. To confirm the requirement for P_4 _signaling in embryoblast development, hESC colonies just before entering the EB stage were treated with or without the PR antagonist RU-486 [37]. When compared with controls, colonies treated with RU-486 failed to form cystic structures (cavitation) and instead formed solid irregular spheres (Figure 4c) that did not express nestin (see Supplemental figure S3 in Additional file 1).

We next examined the requirement for P_4 _signaling during neurulation. hESC colonies grown into a pre-EB stage were treated with either P_4_, RU-486, P_4 _+ RU-486, or neither by using an especially made medium containing all ingredients except P_4_. In the presence of P_4_, control rosettes displayed a minimum of three rosette structures inside of the cavity (Figure 4c(iii)). Compared with controls, pre-EBs treated without P_4 _(Figure 4c(v))or with RU-486 (Figure 4c(iv)) retained a spherical shape but failed to form rosettes with columnar neuroectodermal cells after 17 days in culture. Morphologic changes were more severe in the absence of P_4 _than with RU-486. That neuroectoderm failed to form was confirmed by the absence of nestin expression in RU-486-treated compared with P_4_-treated pre-EBs (Figure 4d). These results confirm our previously reported findings that P_4 _is the differentiation factor present in the neural induction media (N2 component) essential for neuroectodermal rosettes formation [4]. These results therefore suggest an obligatory role for P_4 _signaling in gastrulation and neurulation during early embryogenesis.

hCG/LH signaling via the LHCGR increases hESC proliferation, but also P_4 _synthesis that decreases cell proliferation and promotes differentiation. At what point these functions bifurcate, and what other factors regulate hCG-induced proliferation versus hCG-induced P_4_-mediated differentiation (for example, BMP signaling) remain to be determined. Interestingly, hESCs default toward a primitive neural stem cell fate if maintained for any length of time in culture [38]. Because hESCs express gonadotropins (Figure 1b), and hCG signaling promotes P_4 _production (Figure 2b), which induces lineage commitment toward a neuroectodermal phenotype (Figures 4b, c), we tested whether hCG might act to differentiate hESCs toward a neuronal lineage. hCG treatment induced nestin expression (205-kDa variant) in hESCs (Figure 4e), indicating that endogenous gonadotropin production by hESCs (Figure 1) or trophoblastic cells [3] may be sufficient for NPC formation, thereby explaining the *extrinsic *hormonal signals regulating the 'default pathway' of hESC differentiation into neuronal lineages [38].

## Discussion

These results indicate that trophoblastic hCG production adjacent to the embryoblast is required not only for trophoblast steroidogenesis and attachment to the uterine wall, but also for signaling normal growth and development of the embryoblast. hCG has known growth and differentiation functions during pregnancy, in which extravillous invasive cytotrophoblast cells [39,40] or nonvillous cytotrophoblast cells [41,42] secrete hyperglycosylated hCG that acts in an autocrine/paracrine manner to promote invasion [40,43]. Hyperglycosylated hCG-induced invasion of extravillous cytotrophoblast cells is critical for successful pregnancy implantation [44]. Hyperglycosylated hCG also promotes growth and invasion of choriocarcinoma cells across membranes *in vitro *and promotes extensive invasion, growth, and malignancy of choriocarcinoma cells transplanted into nude mice *in vivo *[39,40]. Regular hCG also has growth properties, promoting the induction of neoangiogenesis during endometrial vascularization [45,46]. Moreover, the free glycoprotein α-subunit of gonadotropins has been shown to stimulate differentiation of prolactin cells in the pituitary [47] and endometrial stromal cell decidualization in the placenta [48]. As recently commented on, these well-described growth and malignancy properties of hCG have been largely neglected [49,50]. In our experiments, blocking LHCGR signaling with antisense and a specific blocking antibody suppressed hESC growth (Figure 2) strongly supports a role of hCG in promoting embryoblast growth. Although regular hCG did not increase cell proliferation above that of media containing no growth factors (Figure 1f), it is likely that either regular or glycosylated forms of hCG are the mitogenic factor promoting hESC division.

Although the structural importance of P_4 _and alloprogesterone has previously been recognized by its early synthesis (by at least day 13) within the developing rat central nervous system [51], our results demonstrate an earlier (within the first 7 days) and absolute requirement for P_4 _during both EB and neuroectodermal rosette differentiation, as indicated by the findings that (a) PR-A is expressed by hESCs and EBs, (b) RU-486 prevents normal cavitation of hESC colonies and columnar neuroectodermal rosette formation, (c) RU-486 prevents nestin expression, (d) P_4 _induces nestin expression in hESC, (e) P_4 _withdrawal from pre-EBs inhibits neuroectodermal rosette formation, and (f) hCG upregulates StAR processing for cholesterol transport and P_4 _synthesis and secretion. Extrapolation of these results from this model system would suggest that paracrine/juxtacrine signaling of hCG for mobilization of cholesterol for P_4 _production by the embryoblast/syncytiotrophoblast after conception is essential for blastulation and neurulation. The P_4_-induced differentiation of hESCs into neuroectodermal cells may be mediated by the alternate processing of the AβPP, because AβPP processing toward the nonamyloidogenic pathway after the addition of P_4 _(neural induction media) is required for neural precursor cell formation [18].

Progestogens are demonstrated neurotrophic agents that promote adult neurogenesis, neuronal survival, and neuroprotection [34,35,52-58]. P_4 _is necessary and sufficient (in neurobasal media) for the maintenance and differentiation of primary hippocampal/cortical/striatal neurons *in vitro *[34]. That P_4 _is the hormone regulating these key early developmental events is consistent with its location high in the steroid synthetic pathway; P_4 _is the first steroid synthesized from pregnenolone, the precursor to all other steroids. Moreover, the expression of PR-A by hESCs and EBs, and the findings that PR-A expression is not altered by either agonists or antagonists suggests an important function for PR-A signaling early in embryogenesis. The potential for P_4 _to regulate organogenesis has been reported during puberty and adulthood, in which P_4 _is obligatory for the development of the tertiary ducts on the mammary gland, and the physiological differentiation of the lobuloalveolar system from the lobular buds [59]. In the adult, P_4 _also has been demonstrated to regulate bone formation [60], promote angiogenesis and arteriogenesis [61], and promote formation of the placenta.

Support for LH in mediating neurogenesis has been reported in mice, in which LH induces neurogenesis in the adult hippocampus [62]. Whether these properties of LH/hCG in the adult brain are mediated via P_4_, as we demonstrated for hESCs (Figures 1 through 4) remains to be determined, although progestogens have been shown to increase significantly rat neuroprogenitor cell and human neural stem cell proliferation [35,63], and to promote neurite development and migration that lead to changes in synaptogenesis [64-67]. The autocrine/paracrine signaling of hCG for P_4 _synthesis within the blastocyst is consistent with the recent findings that a 'mini-HPG' axis appears within the extrahypothalamic/pituitary brain that serves to regulate brain neurosteroid production [68]. Identification of other members (hormones and hormone receptors) of the HPG axis that regulate hCG/LH production, for example, might be predicted.

The requirement for P_4 _during cavitation processes indicates the structural influence of these molecular pathways on the developing embryo within the first 7 days, but also on the formation of the neural tube at around day 17 to 19, which will influence future neural connectivity. The suppression of P_4 _signaling at this time (for example, with RU-486) [69] will block these time-sensitive developmental processes. Given that the relative binding affinity of RU-486 for the PR is twice that of P_4 _[70], and that treatment of hESC with 20 μ*M *RU-486 in our study (Figures 3 and 4) is equivalent to the dosage used for the termination of pregnancies (~6-19 μ*M) *[37], administration of RU-486 would clearly have a negative effect on the developing embryo. Thus, the abortifacient effects of RU-486 in blocking PR signaling therefore also extend to blocking blastulation and neurulation and the normal growth and development of the embryo.

The maintenance of hESCs in a pluripotent state in culture is dependent on the presence of a member of the FGF family as well as a member of the TGF-β superfamily [25]. Whether FGF and TGF-β also are physiologic signals produced by trophoblastic cells that signal the embryoblast is unknown, although FGF and activins have known growth and differentiation properties [13,71].

**Figure 5:**
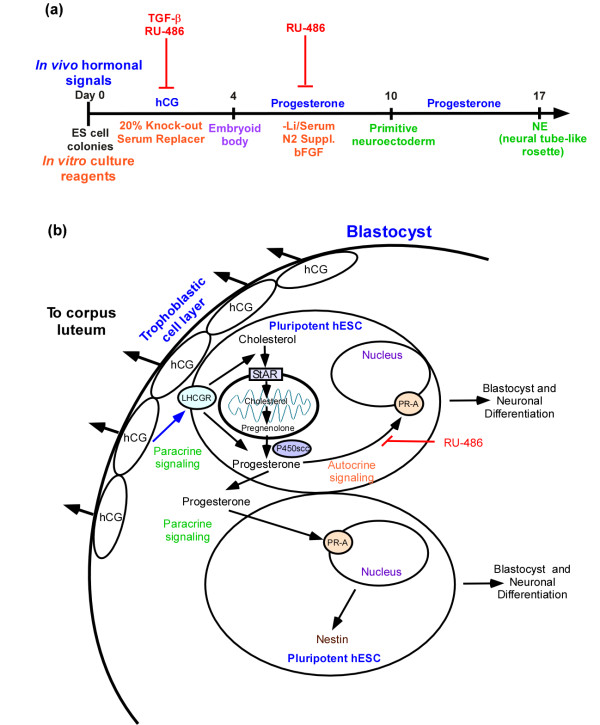
**Model of the spatiotemporal autocrine and paracrine pathways regulating blastulation and neurulation**. Based on our current knowledge and the data presented in this article regarding the hormonal regulation of early embryogenesis, we present a model of **(a) **the time course for the induction of embryoid bodies and neuroectodermal rosettes by hCG and P_4_, and **(b) **putative autocrine and endocrine signaling pathways involved in cell proliferation, steroidogenesis, and differentiation of the blastocyst and primitive neural tube.

We present a model of the time course for *in vitro *differentiation of hESCs into an EB and into a neuroectodermal rosette with the physiological hormones hCG and P_4 _(Figure 5a), and of the autocrine and paracrine pathways within the blastocyst that might operate to regulate blastocyst and neuroectodermal development (Figure 5b). Our data suggest that trophoblastic hCG production adjacent to the embryoblast could be required, not only for trophoblast steroidogenesis and attachment of the blastocyst to the uterine wall [72], but also for signaling normal proliferation and differentiation of the embryoblast. hCG-induced P_4 _synthesis therefore has, in addition to its role in uterine decidualization for the implantation and maintenance of pregnancy, an obligatory role before the formation of neural precursor cells (blastulation), as well as an inductive role in the directed differentiation and specification of the first neuronal cell types (organogenesis; Figure 4) and the formation of the neural tube. If future *in vivo *studies should confirm these *in vitro *results, this paracrine/juxtacrine signaling by extraembryonic tissues would be the commencement of trophic support by placental tissues in the growth and development of the human embryo.

## Conclusions

This work demonstrates that hCG signaling via the LHCGR induces hESC proliferation. Differentiation of hESCs into neural precursor cells was mediated by the upregulation of steroidogenesis by hCG signaling through the LHCGR. Suppression of progesterone (P_4_) signaling inhibited EB and neuroectodermal rosette formation, suggesting an obligatory role for this steroidogenic pathway during hESC differentiation. Together our results suggest that pregnancy-associated hCG and P_4 _could be physiologic signals that promote proliferation and differentiation during blastulation and neurulation.

## Abbreviations

E_2_: 17β-estradiol; EBs: embryoid bodies; EIA: enzyme immunoassay; hCG: human chorionic gonadotropin; hESCs: human embryonic stem cells; LHCGR: luteinizing hormone/chorionic gonadotropin receptor; NPCs: neural precursor cells; P_4_: progesterone; PR-A: P_4 _receptor-A; StAR: steroid acute regulatory protein.

## Competing interests

The authors declare that they have no competing interests.

## Authors' contributions

MJG maintained the hESCs, designed and performed the experiments, analyzed the data, interpreted the results and helped write the manuscript. PP maintained the hESCs, performed the experiments, and analyzed the data. MMK maintained the hESCs. RLB helped conceive the hCG experiments. SVM performed the experiments on PR, interpreted results, and helped write the manuscript. CSA conceived the study, participated in its design and coordination, interpreted the data, and wrote the manuscript.

## Supplementary Material

Additional file 1 **Additional data**. LHCGR mRNA expression in hESCs (Supplemental figure S1); LH and hCG regulation of Oct-3/4 expression in hESC (Supplemental figure S2); progesterone regulation of nestin expression in EBs and rosettes (Supplemental figure S3). Click here for file
