# Supplementary material for: The pregnancy hormones human chorionic gonadotropin and progesterone induce human embryonic stem cell proliferation and differentiation into neuroectodermal rosettes
Source: Stem Cell Res Ther. 2010 Sep 13;1(4):28. doi: 10.1186/scrt28 (PMC2983441; doi:10.1186/scrt28)

**Supplemental Data**

**Figure 1:** Total RNA extracted from H9 hESC cells was amplified via RT-PCR using sequence specific primers for LHCGR. The expected 462pb band was observed. A molecular weight ladder is shown on the left.

**Figure 2:** Equal amounts of protein from cell lysates of hESC grown in mTeSR1 media and treated with LH (5, 10, and 100 mIU/mL) or hCG (50, 500, and 5,000 mIU/mL) for 6 d were analyzed by immunoblot with an antibody against Oct-3/4 expression as described in Fig. 1.

**Figure 3:** hESC colonies were placed into EB media (containing serum) in the absence (control) or presence of RU-486 (20 µM) and rocked gently for an additional 10 d to allow EB formation. EBs were incubated in neural induction media containing no P4, P4 (2 µM) or RU-486 (20 µM) for 11 days. The structures were then collected and equal amounts of protein from cell lysates analyzed for nestin by immunoblot analysis. RU-486 suppressed nestin expression and both EB and rosette formation.


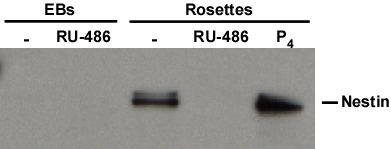

Supplement: Additional file 1 — Additional data. LHCGR mRNA expression in hESCs (Supplemental figure S1); LH and hCG regulation of Oct-3/4 expression in hESC (Supplemental figure S2); progesterone regulation of nestin expression in EBs and rosettes (Supplemental figure S3). [file scrt28-S1.DOC]
